# Supplementary material for: Transcriptome and Metabolome Analyses Revealed the Response Mechanism of Sugar Beet to Salt Stress of Different Durations
Source: Int J Mol Sci. 2022 Aug 24;23(17):9599. doi: 10.3390/ijms23179599 (PMC9455719; doi:10.3390/ijms23179599)
Supplement: Supplementary file 1 [file ijms-23-09599-s001.zip › Figure S1 The changes of sugar beet O68 seedlings under different durations of salt treatments.pdf]

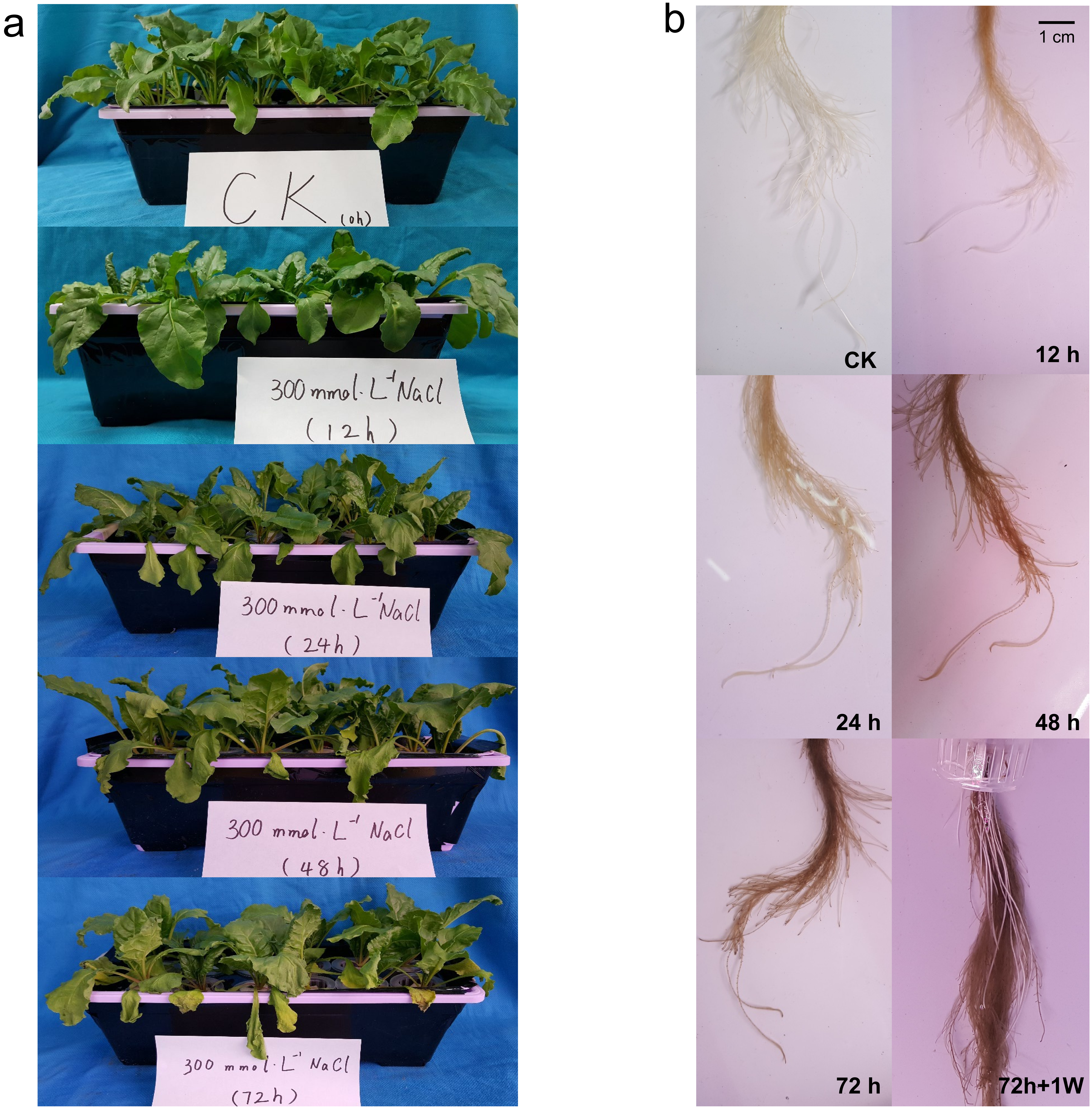

Fig S1. The changes of sugar beet O68 seedlings under different durations of salt treatments. (a) leaves, (b) roots (Photos taken from the same sample); 1 w represents rehydration for one week after 72 h salt treatment.
