# Supplementary material for: Transcriptome and Metabolome Analyses Revealed the Response Mechanism of Sugar Beet to Salt Stress of Different Durations
Source: Int J Mol Sci. 2022 Aug 24;23(17):9599. doi: 10.3390/ijms23179599 (PMC9455719; doi:10.3390/ijms23179599)
Supplement: Supplementary file 1 [file ijms-23-09599-s001.zip › Figure S2 Overview of gene expression patterns in each sample under salt stress.pdf]

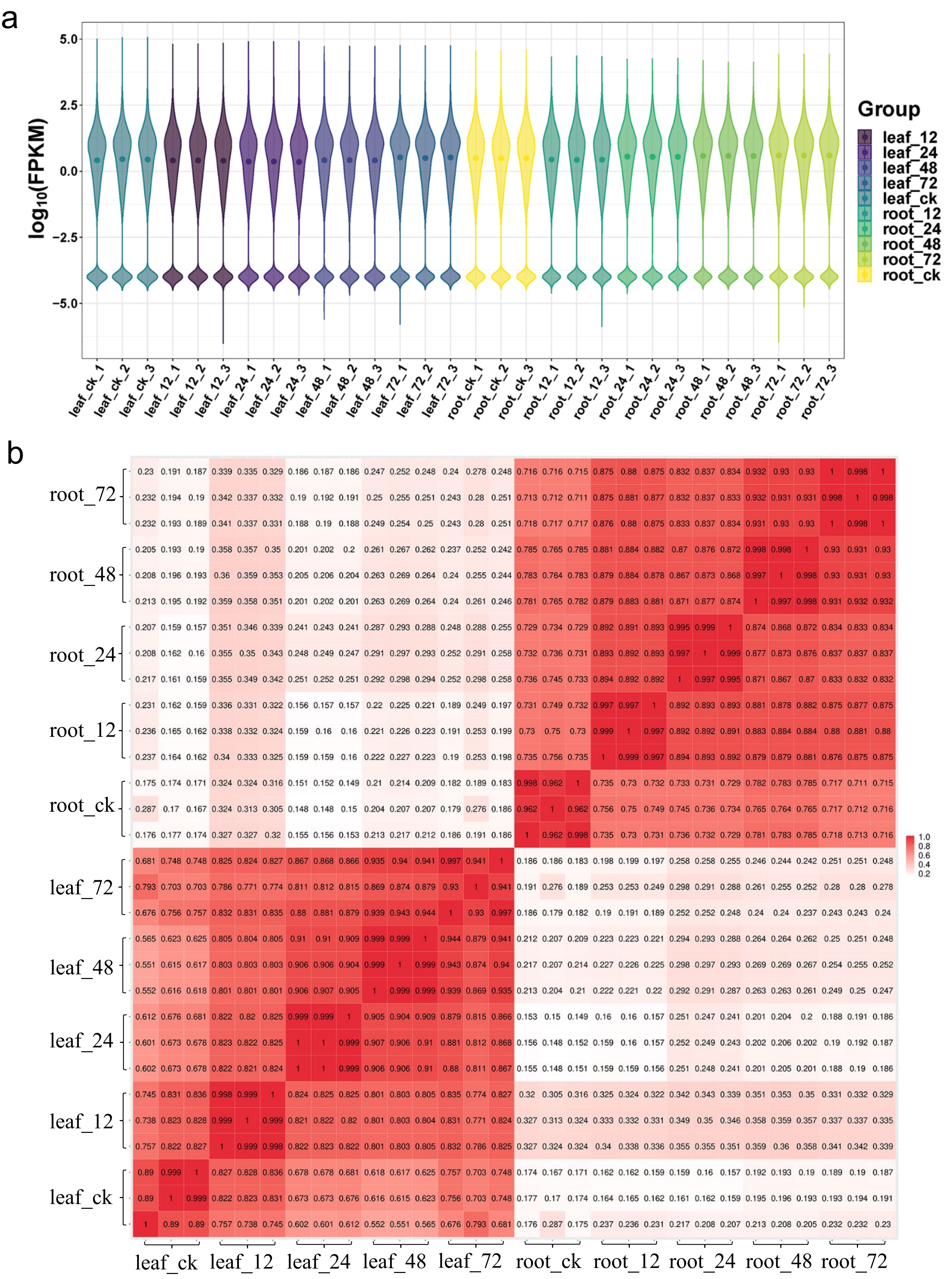


**Fig.S2 Overview of gene expression patterns in each sample under salt stress.** (a) Gene expression levels (b) Correlation of expressed genes in each sample.
