# Supplementary material for: Transcriptome and Metabolome Analyses Revealed the Response Mechanism of Sugar Beet to Salt Stress of Different Durations
Source: Int J Mol Sci. 2022 Aug 24;23(17):9599. doi: 10.3390/ijms23179599 (PMC9455719; doi:10.3390/ijms23179599)
Supplement: Supplementary file 1 [file ijms-23-09599-s001.zip › Figure S5 Construction of gene co-expression networks.pdf]

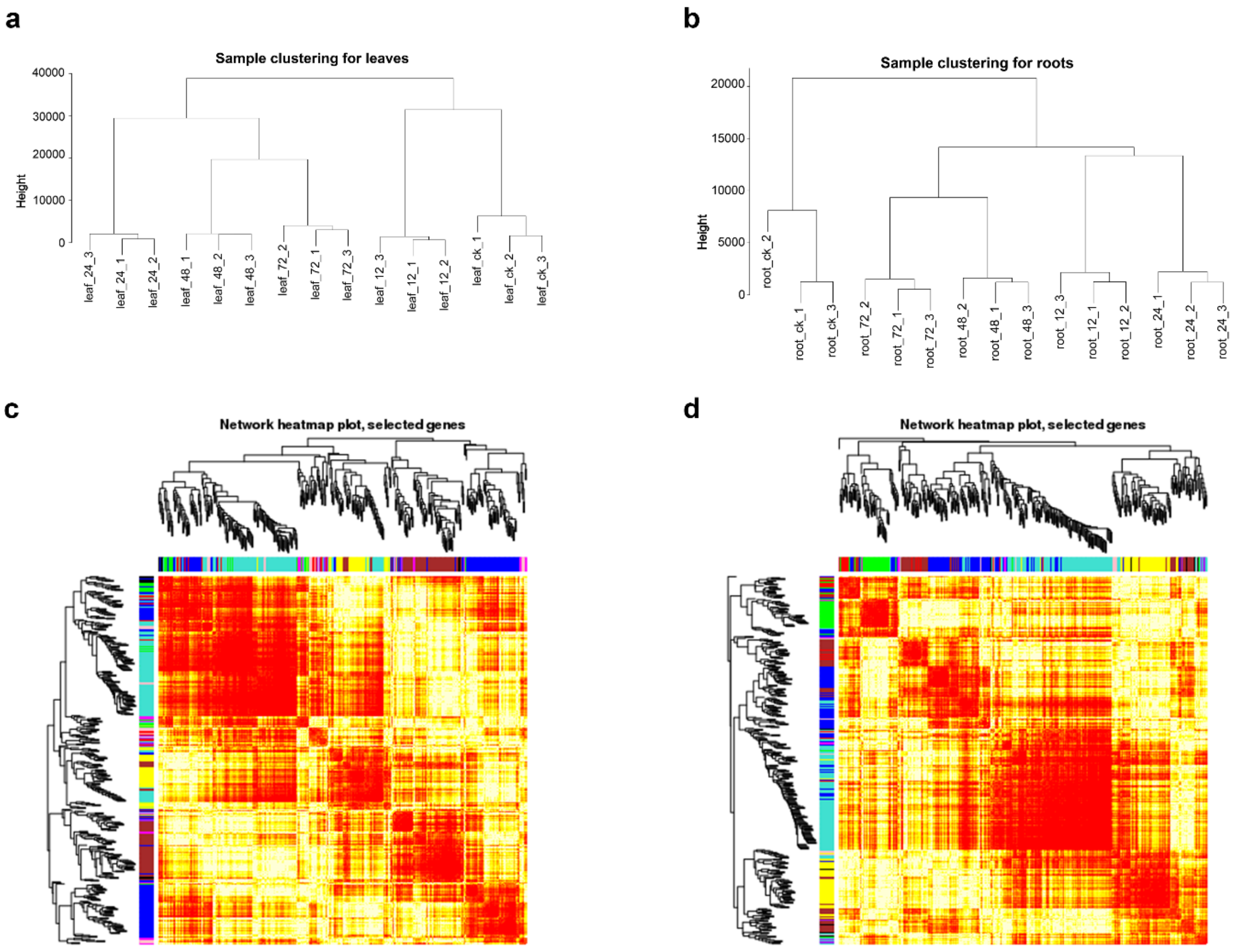

Fig. S5 Construction of gene co-expression networks. Sample outlier check for leaves (a) and roots (b). Network heatmap plot of hierarchical clustering of the TOM matrix for the co-expressed genes in leaves (c) and roots(d).
