# Supplementary material for: Transcriptome and Metabolome Analyses Revealed the Response Mechanism of Sugar Beet to Salt Stress of Different Durations
Source: Int J Mol Sci. 2022 Aug 24;23(17):9599. doi: 10.3390/ijms23179599 (PMC9455719; doi:10.3390/ijms23179599)
Supplement: Supplementary file 1 [file ijms-23-09599-s001.zip › Figure S7 Principal Component Analysis (PCA) and partial least squares discrimination analysis (PLS-DA) of metabolites between st and ck.pdf]

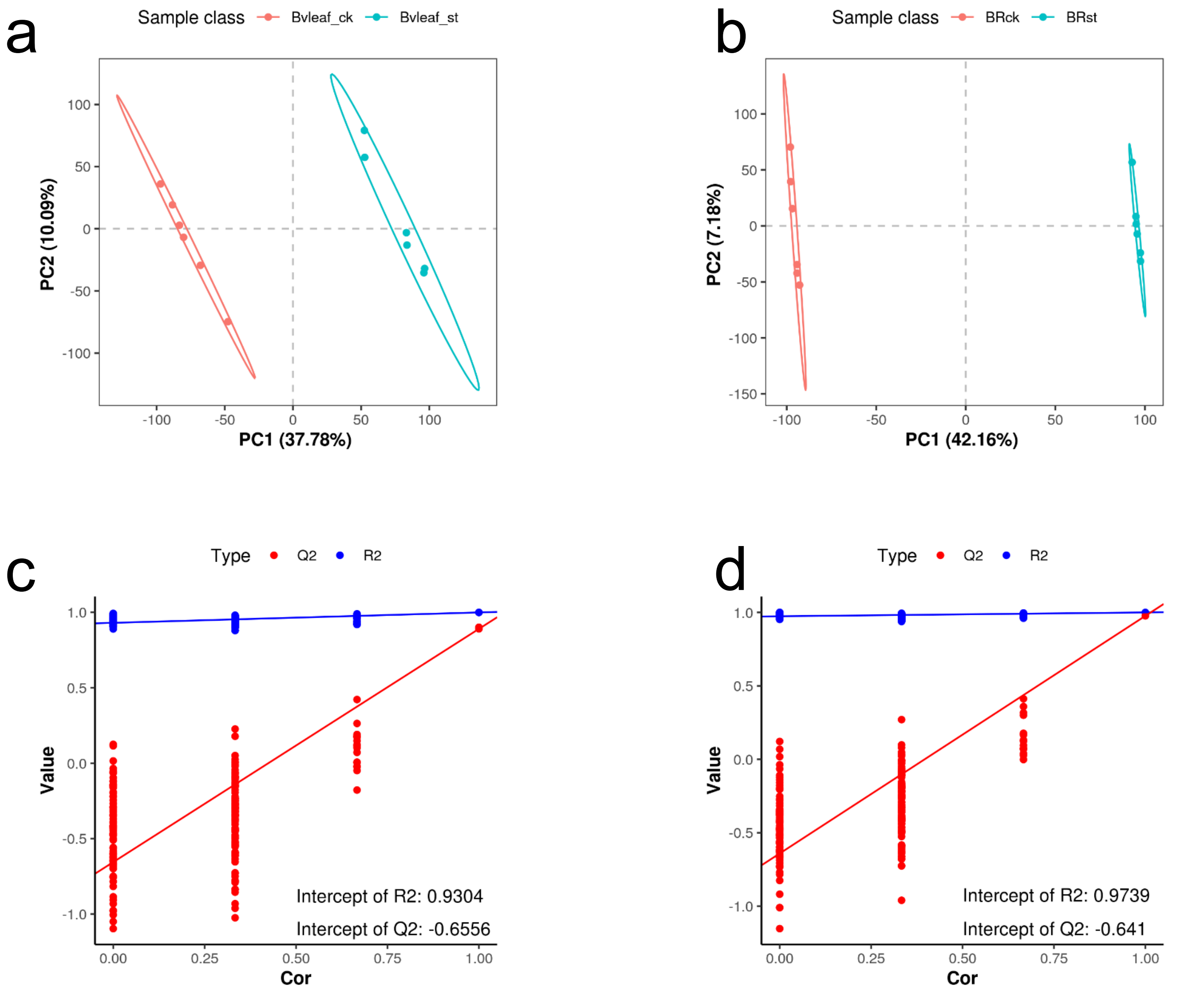

Fig.S7 Principal Component Analysis (PCA) and partial least squares discrimination analysis (PLS-DA) of metabolites between st and ck. PCA of leaves (a) and roots (b). PLS-DA of leaves (c) and roots (d). R2 represents model explanation rate, Q2 represents model prediction rate.
