# Supplementary figures and images for: Transcriptome and Metabolome Analyses Revealed the Response Mechanism of Sugar Beet to Salt Stress of Different Durations
Source: Int J Mol Sci. 2022 Aug 24;23(17):9599. doi: 10.3390/ijms23179599 (PMC9455719; doi:10.3390/ijms23179599)

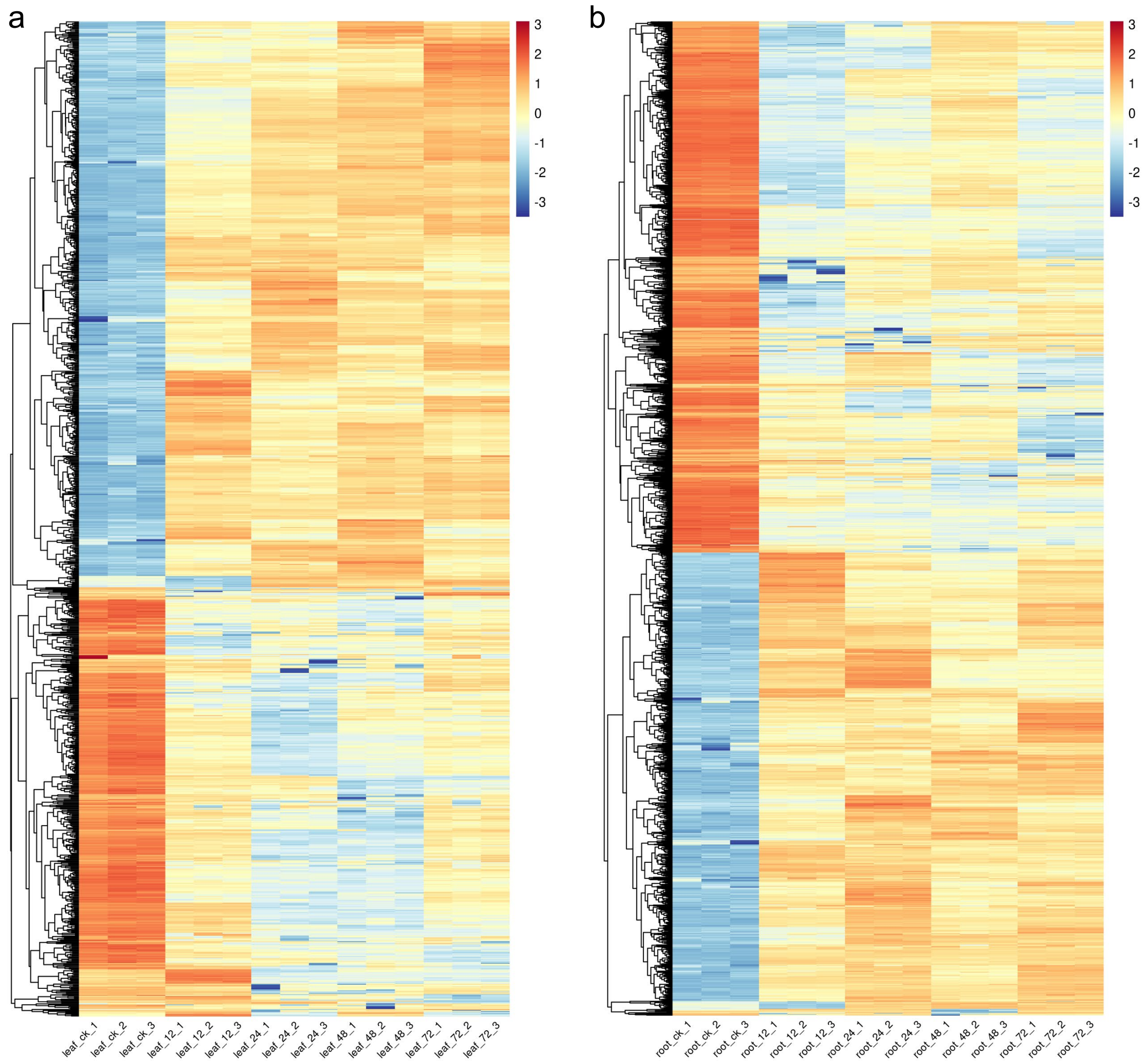

**Fig.S3 Expression patterns of common DEGs. (a)leaves (b)roots.**

Supplement: Supplementary file 1 [file ijms-23-09599-s001.zip › Figure S3 Expression patterns of common DEGs.pdf]

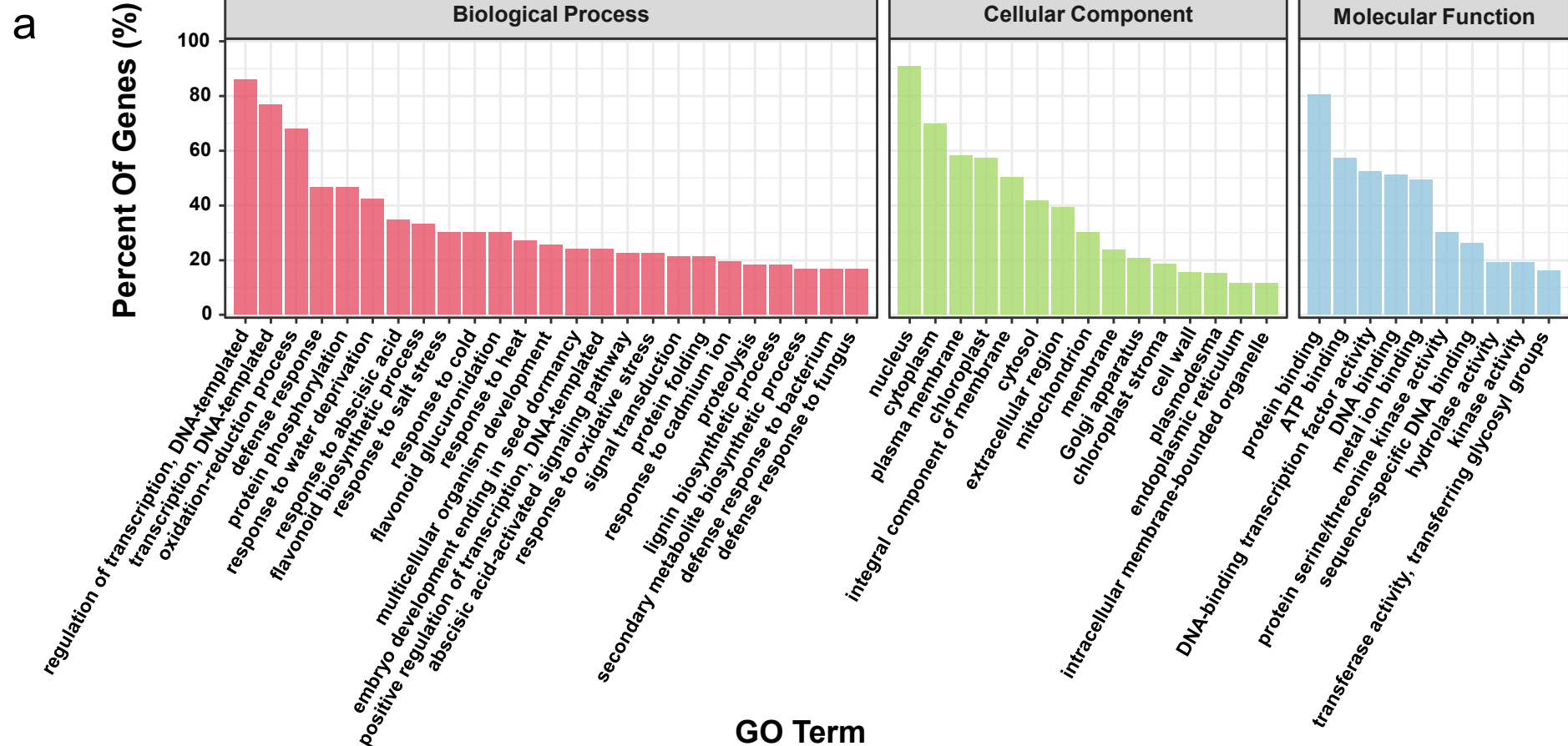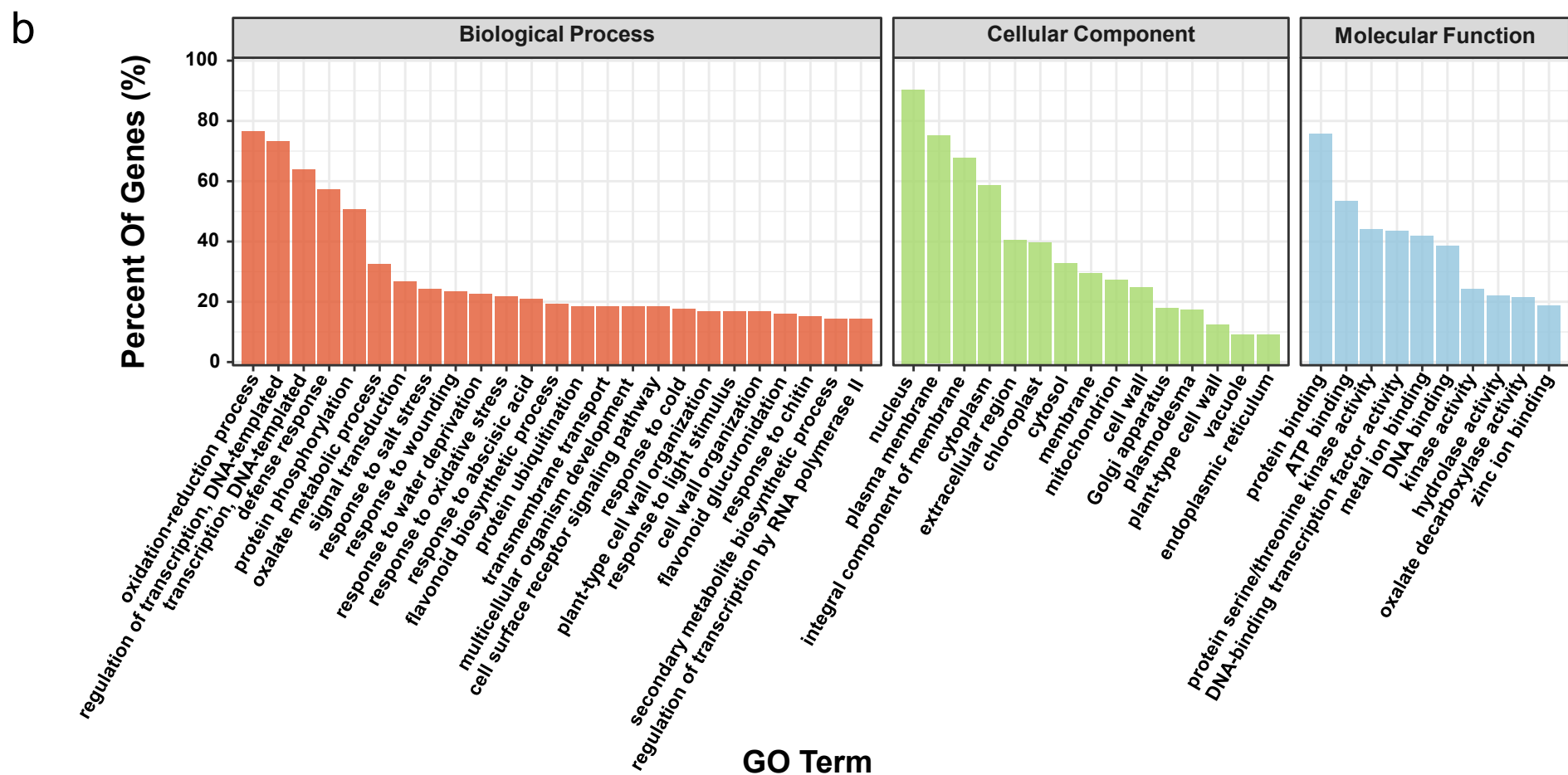

**Fig.S4 GO annotation of common DEGs in leaves (a) and roots (b).**

Supplement: Supplementary file 1 [file ijms-23-09599-s001.zip › Figure S4 GO annotation of common DEGs in leaves(a) and roots(b).pdf]

a

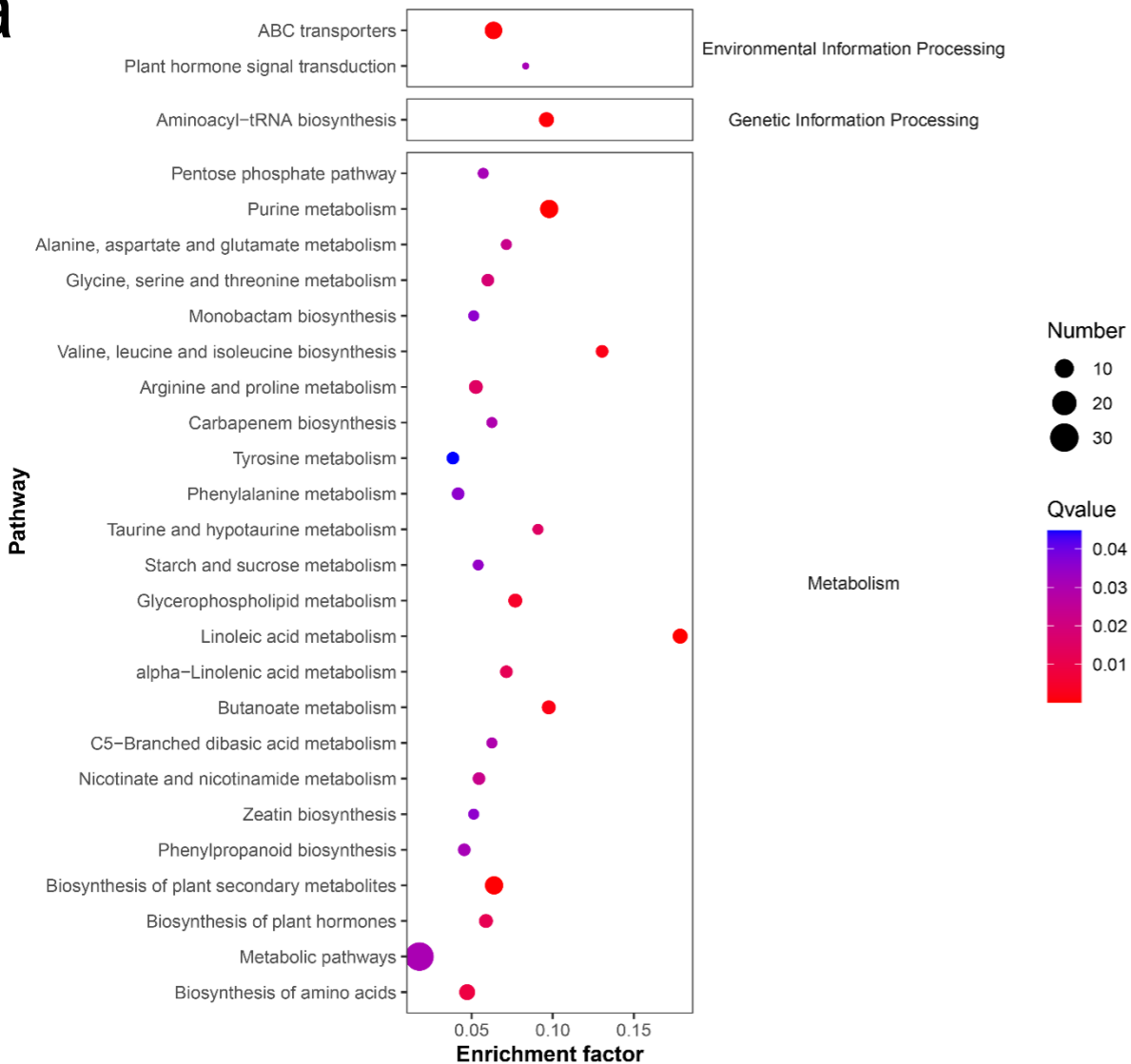

b

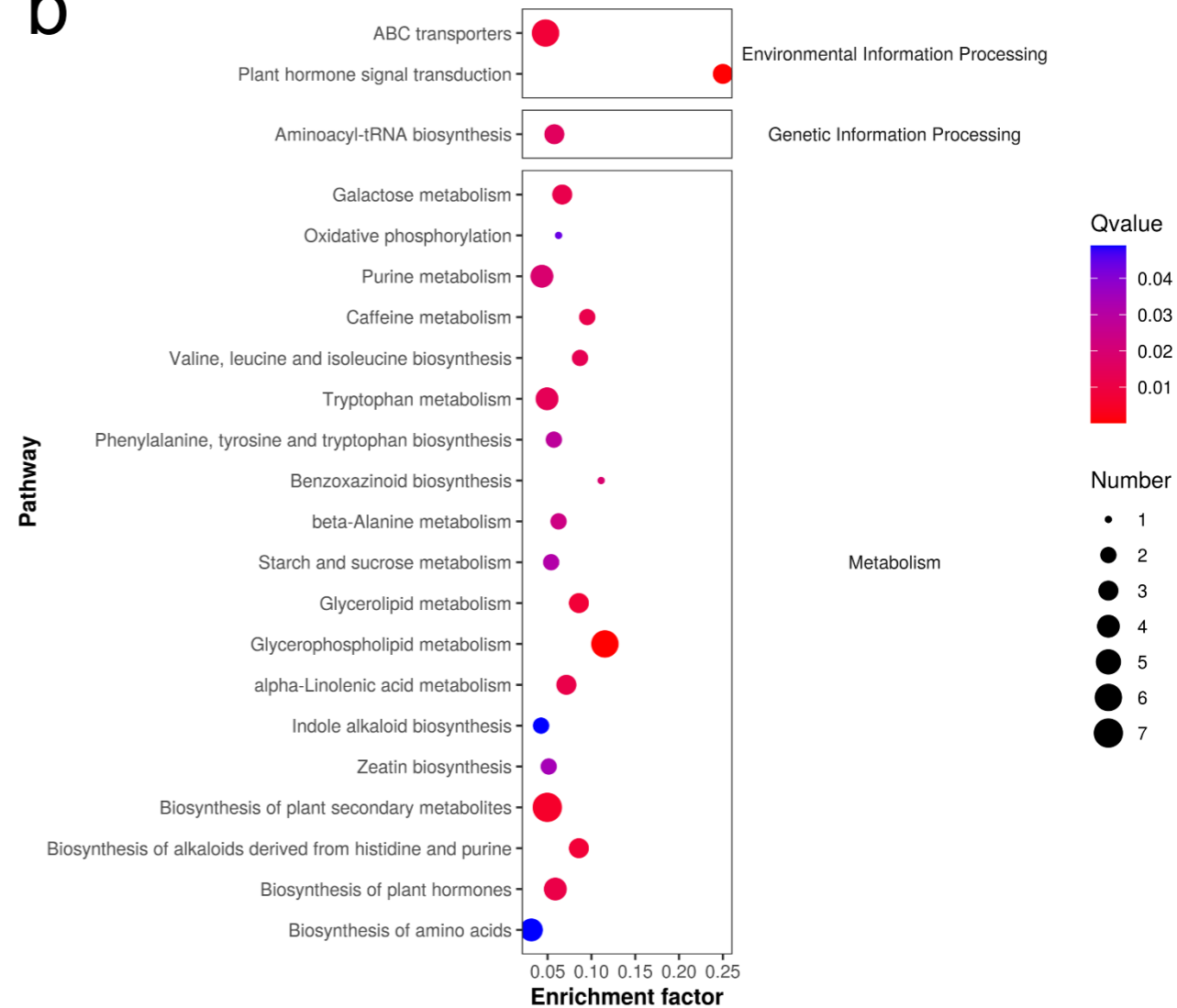

Fig.S8 KEGG annotation of DEMs in leaves(a) and roots(b).

Supplement: Supplementary file 1 [file ijms-23-09599-s001.zip › Figure S8 KEGG annotation of DEMs in leaves(a) and roots(b).pdf]
